# Tackling Faults in the Industry 4.0 Era—A Survey of Machine-Learning Solutions and Key Aspects

**DOI:** 10.3390/s20010109

**Published:** 2019-12-23

**Authors:** Angelos Angelopoulos, Emmanouel T. Michailidis, Nikolaos Nomikos, Panagiotis Trakadas, Antonis Hatziefremidis, Stamatis Voliotis, Theodore Zahariadis

**Affiliations:** 1 General Department, National and Kapodistrian University of Athens, Thesi skliro, Psahna, 34400 Evia, Greece; aaggelopoulos@teiste.gr (A.A.); ptrakadas@uoa.gr (P.T.); ahatzie@uoa.gr (A.H.); svoliotis@uoa.gr (S.V.); zahariad@uoa.gr (T.Z.); 2 Telecommunications, Signal Processing and Intelligent Systems (TelSiP) Research Laboratory, Department of Electrical and Electronics Engineering, School of Engineering, University of West Attica, Ancient Olive Grove Campus, 12244 Aigaleo, Greece; emichail@uniwa.gr; 3 Department of Information and Communication Systems Engineering, School of Engineering, University of the Aegean, 83200 Samos, Greece

**Keywords:** Industry 4.0, machine learning, fault detection, predictive maintenance, security, anomaly detection, human–machine interaction

## Abstract

The recent advancements in the fields of artificial intelligence (AI) and machine learning (ML) have affected several research fields, leading to improvements that could not have been possible with conventional optimization techniques. Among the sectors where AI/ML enables a plethora of opportunities, industrial manufacturing can expect significant gains from the increased process automation. At the same time, the introduction of the Industrial Internet of Things (IIoT), providing improved wireless connectivity for real-time manufacturing data collection and processing, has resulted in the culmination of the fourth industrial revolution, also known as Industry 4.0. In this survey, we focus on the vital processes of fault detection, prediction and prevention in Industry 4.0 and present recent developments in ML-based solutions. We start by examining various proposed cloud/fog/edge architectures, highlighting their importance for acquiring manufacturing data in order to train the ML algorithms. In addition, as faults might also occur from sources beyond machine degradation, the potential of ML in safeguarding cyber-security is thoroughly discussed. Moreover, a major concern in the Industry 4.0 ecosystem is the role of human operators and workers. Towards this end, a detailed overview of ML-based human–machine interaction techniques is provided, allowing humans to be in-the-loop of the manufacturing processes in a symbiotic manner with minimal errors. Finally, open issues in these relevant fields are given, stimulating further research.

## 1. Introduction

Mankind has made significant advancements during the last 300 years, in the area of industrial manufacturing. The first industrial revolution focused on mechanical innovations relying on steam and water, while the second one leveraged electrification and advanced machine tools, further boosting and improving the production output. Then, starting from the 1950s, the third industrial revolution adopted increased digitization using semi-conductors and more recently, communication networks, paving the way for automated manufacturing. During the last decade, artificial intelligence (AI) and machine learning (ML) have been introduced in the manufacturing sector, enabling more efficient processes, sustainability with reduced waste and consumption of materials, safer working environments and increased quality and productivity. AI/ML-based manufacturing can offer various manufacturing innovations by providing fault detection and prediction, optimal use of raw materials and resources, exploiting the heterogeneous big data analysis and the interconnected manufacturing plants [1,2,3,4,5].

### 1.1. Industry 4.0

The fourth industrial revolution (Industry 4.0) aims at providing an industrial environment for real-time, intelligent, interoperable, and autonomous manufacturing environments [6]. In order to realize this vision, Industry 4.0 is based on recent innovative information and communication technologies, such as cyber-physical systems (CPS), Internet of Things (IoT) and cloud computing (CC) [7], having attracted the research interest of the academia and the industry in a plethora of applications [8]. CPS results in the interconnection of physical elements with cyber-elements [9]. More specifically, physical elements comprise components such as sensors, operator panels and computers, collaborating and communicating to collect and provide data to cyber-elements where management, processing and decision-making takes place [4,9]. Furthermore, IoT enables the real-time interconnection of different objects, for example, sensors, actuators, machines and robots among others, in a safe and reliable manner. At the heart of IoT lie heterogeneous communication networks, including fifth generation (5G) networks, Wi-Fi, machine-to-machine (M2M) deployments and cloud technologies (cloud, fog, edge) [10,11,12]. At the same time, humans should maintain an important role in the Industry 4.0 environments, empowered with smart devices, virtual and augmented reality (VR/AR), being in the loop of the manufacturing process and taking advantage of the AI/ML-based decision-making [13]. Thus, considering these advanced technologies, Industry 4.0 promises to radically change the current industrial production processes benefiting industrial stakeholders, personnel and consumers, promoting environmental sustainability.

In the context of Industry 4.0, a plethora of applications is envisioned, providing flexibility, competency, real-time self-optimization, and automation, as well as accomplishing complex tasks and satisfying strict quality requirements in intertwined digital and physical procedures [8]. More importantly, the relevant ubiquitous applications mainly span in the manufacturing and production development areas [14]. To evolve these applications, fault detection, prediction and prevention play a major role. With the contribution of ML algorithms, early and accurate fault detection can lead to minimum downtime, owing to the recognition of damaged and defected products or parts, in real-time. The wealth of data contributes to accurate prediction of machine condition, remaining useful life (RUL), and faults, leading to an appropriate and cost-effective maintenance schedule, thus minimizing the downtime, due to a fault occurrence [10,15]. The human factor has also an important role in the production procedure of the Industry 4.0 ecosystem, whereas collaboration with robots and machines is a critical challenge within factory halls. Human activity recognition and decision-making algorithms increase operators’ performance, leading to efficient and safe production, as well as to the minimization of ramp-up time.

### 1.2. Machine Learning

The application of ML algorithms requires the existence of a vast amount of data to trigger decision-making in several industrial processes. In this regard, the implementation of novel technological paradigms, such as CPS and IoT, enables the generation of different types of data structures, as it has been observed in works focusing on Big Data Analytics (BDA) [15,16,17]. In general, data has a specific life cycle (source, collection, storage, processing, visualization, transmission, application) [18]. However, most of the time, the gathered data that will be processed in subsequent steps, is confused with noisy data generated from the surrounding environment, making it difficult to separate the original data set from noise. On the other hand, fast changing dynamic environments and different machine working states impose significant challenges to ML-based fault detection, prediction and prevention. Overall, the need for reliable and accurate real-time transmission and computation arises, while security issues are becoming increasingly serious, due to the increased level of interconnection among the different subsystems [10].

The ML algorithms can be categorised into supervised learning, unsupervised learning, reinforcement learning (RL) and deep learning (DL) algorithms [15]. Each category is briefly described below.

- **Supervised learning** is a method where an expert inserts known outputs for specific inputs to train the algorithm and is widely used for classification and regression [15,19,20]. Thus, supervised ML is usually employed in scenarios with labeled data availability. Popular algorithms include artificial neural networks (ANNs) and support vector machines (SVMs) [15,19,21].
- **Unsupervised learning** where there is no feedback provided from anyone and the algorithm finds patterns in unknown data sets (clustering, association rules, self-organised maps) [15,19,21] and so, unlabeled data are used for training purposes. The most popular and well-known unsupervised algorithm is principal component analysis (PCA), mainly used for monitoring purposes [19].
- **Reinforcement learning** refers to unsupervised ML operation, examining if a chosen action resulted in a reward, for a specific performance metric [15]. RL demands sequential actions and tries their outcome, selecting those better fitting the problem at hand. So, RL significantly departs from other learning categories which are based on leveraging historical data, creating intelligence from previous decisions and rewards.
- **Deep learning** where multiple layers have been employed in order to build an ANN, which is able to make intelligent decisions, handling large amounts of data with high complexity, without any human intervention [15,22,23]. Some DL algorithms are convolutional neural networks (CNNs), restricted Boltzmann machine (RBM) and auto-encoders (AE) [23].

**Figure 1:**
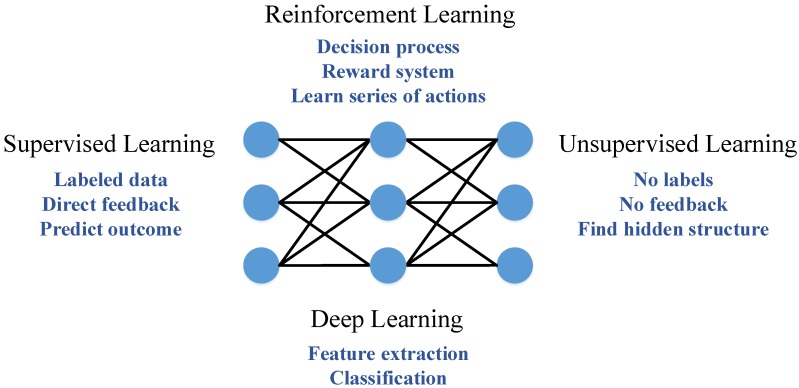
Various machine learning (ML) categories and their key characteristics.

In Figure 1, the different ML categories are summarised and their key characteristics are highlighted. It is evident that as the Industry 4.0 era is upon us, there exists an ever increasing adoption level of ML algorithms to satisfy the needs of different aspects of industrial settings. These include process monitoring and quality control, fault detection and diagnosis, as well as machine health monitoring and predictive maintenance [24]. Moreover, the capabilities of ML, regarding the timely processing of an abundance of data are critical to safeguarding the cyber-security of the Industrial Internet of Things (IIoT) enabled interconnected manufacturing environments, accurately detecting and mitigating threats [22,25].

### 1.3. Previous Survey Works

The research area of Industry 4.0 has received several contributions in recent years. The survey in Reference [10] presented an overview of CPS in IIoT settings, giving in detail a relevant architecture, enabling the control of systems and processes, while ML was presented as a promising solution for CPS. Then, the authors of Reference [16] provided a thorough analysis of the role of BDA in IoT and a taxonomy of ML algorithms in BDA-enabled applications, such as self-maintenance, self-prediction and self-configuration. Furthermore, an overview of ML for manufacturing systems was given in Reference [15], discussing implementation issues, as well as the benefits and drawbacks of different categories of ML algorithms, including supervised, unsupervised and RL algorithms, concluding that currently, supervised ML techniques are most appropriate for smart manufacturing. Next, the work in Reference [19] discussed the use of BDA in the process industry and its dependency on ML. More specifically, several supervised and unsupervised ML methods were presented, suggesting that semi-supervised solutions have the potential to provide better trade-offs among implementation complexity, performance and data requirements. In addition, a detailed survey of big data structure and analytic techniques for CPS were examined in Reference [21]. In this context, descriptive (clustering, correlation) and predictive analytic techniques were evaluated.

In the area of machining processes, the impact of ML solutions was presented in Reference [20]. Several machining cases were listed and a brief presentation of ML-based tool wear monitoring and prediction was included, outlining its potential. Regarding the field of smart manufacturing, the role of DL was investigated in Reference [23]. In greater detail, the evolution of DL and its advantages in processing heterogeneous and highly complex data, compared to conventional ML solutions, as well as various DL-based computational methods for improving manufacturing processes were highlighted. Next, the article in Reference [26] focused on three areas for AI/ML application, that is, faster convergence in environments with partial and intermediate AI/ML integration with a single optimization iteration, deep active learning towards optimal topologies through multiple iterations without neglecting the benefits of exploration-exploitation trade-offs and finally, knowledge-based assistants for improved human–machine interaction (HMI) by translating the optimal topology to a concept design, leveraging relevant metadata of historical expert decision.

The study in Reference [27] examined several works on ML-based optimization in the areas of product quality and process improvement. The interdependencies among the used data, the amount of data, the ML algorithms, the adopted optimisers and the specific production problems were discussed, concluding that often, their correlation is not carefully investigated, thus leading to highly complex models, being trained with insufficient amounts of data, exhibiting overfitting and low interpretability. Next, the authors of Reference [28] introduced research strategies for industrial big data collection in intelligent environments, ontology modeling and deduction methods, as well as predictive diagnostic solutions for production lines, relying on deep neural network (DNN) and DL in cloud-assisted devices with self-organised reconfiguration capabilities.

An overview on anomaly detection on industrial wireless sensor networks (IWSN) was given in Reference [25], comparing ML methods, including K-nearest neighbor (KNN), SVM, ANNs and hybrid schemes. Finally, the study in Reference [29] offered a deep insight on data mining techniques for production management, focusing on production scheduling, quality improvement, defect analysis and fault diagnosis. Still, ML-based solutions were not thoroughly presented, thus leaving a gap in the literature. Table 1 provides the summaries of the relevant surveys and possible AI/ML-based solutions that they might discuss within the Industry 4.0 area.

### 1.4. Contributions

This survey aims to provide a comprehensive overview of ML-based solutions for fault detection, prediction and prevention in Industry 4.0. Currently, limited automation of fault detection, prediction and prevention is observed. This trend necessitates staff training towards efficiently using conventional software diagnostic tools. In this way, manufacturers are dealing with increased costs and suboptimal use of human resources. In addition, such manual methods are incapable of early fault detection, adapting to the dynamicity of fault sources and learning new fault types from the abundance of manufacturing data. At the same time, faults may be caused by external sources, such as human errors or cyber-attacks, and so, this survey discusses ML solutions in the fields of HMI and cyber-security. The presentation of a holistic approach on tackling faults in Industry 4.0 contexts, independently of their sources, either being machine tool wear, cyber-attacks or inefficient human–machine collaboration, as well as the detailed analysis of relevant cloud/fog/edge architectures, significantly differentiates our survey from other recent works. More specifically, the following contributions are given:

- An extended discussion on the proposed cloud/fog/edge architectures to enable the acquisition and processing of heterogeneous data in Industry 4.0 for intelligent monitoring and decision-making in fault detection and prediction is provided.
- A thorough presentation of supervised, unsupervised and deep learning solutions in the fields of fault detection and predictive maintenance is given.
- Cyber-security issues and ML-based solutions are discussed, emphasizing on the gains that they provide compared to conventional techniques.
- ML-based HMI aiming to keep humans-in-the-loop of the manufacturing process is presented.
- Open issues are discussed, highlighting the necessary advancements that should be made to enable the Industry 4.0 and beyond vision.

**Figure 2:**
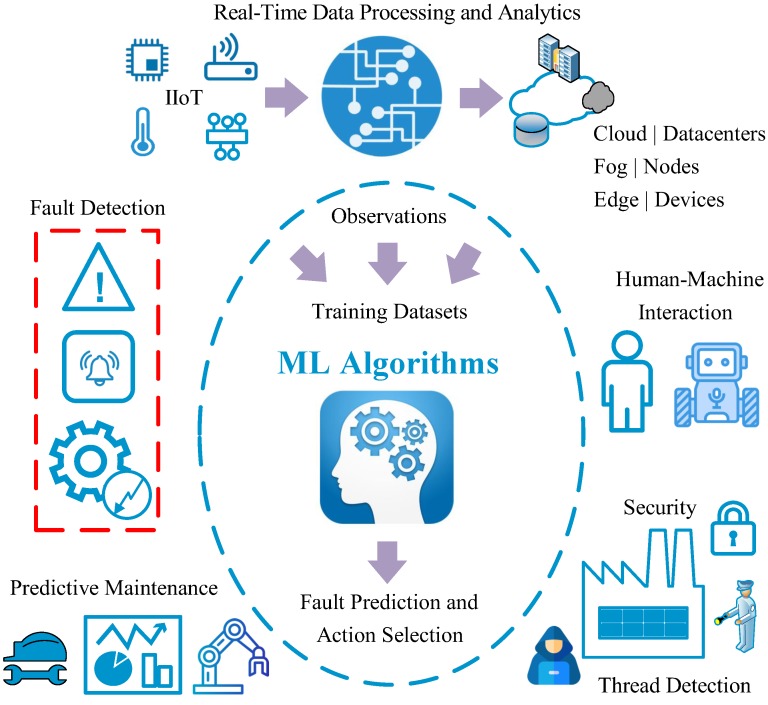
Applications of AI/ML in Industry 4.0 fault detection, prediction and prevention.

Also, in Figure 2, various cases of AI/ML solutions for fault detection, prediction and prevention are depicted. In the following sections, all these areas are thoroughly discussed and details on the impact of ML-based implementations is highlighted.

### 1.5. Structure

**Figure 3:**
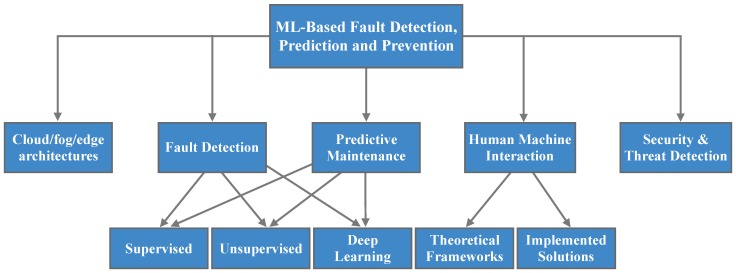
Structure of this survey.

Figure 3 depicts the ML-enhanced aspects in the context of Industry 4.0, being presented in this survey. Stemming from this classification, the rest of the paper is organised as follows. In Section 2, an overview of cloud/fog/edge architectures for the integration of ML for fault detection, prediction and prevention is provided. Then, Section 3 presents and classifies ML techniques tackling fault detection, while Section 4 includes relevant ML solutions for predictive maintenance of machinery and tools. Next, Section 5 highlights the main security and threat detection issues, towards minimizing the impact on the manufacturing processes while Section 6 discusses ML in the context of HMI. Finally, open issues are given in Section 7 and conclusions are drawn in Section 8.

## 2. Cloud/Fog/Edge Architectures

As the IIoT constitutes an emerging network paradigm bridging physical and cyber-entities in CPSs, a combination of interconnected components, cloud and service-oriented computing, as well as real-time analytics can potentially support challenging industrial scenarios and improve productivity in a cost-efficient manner. However, a huge amount of data is typically generated by the sensor devices in particular situations, such as the manufacturing inspection that deals with the detection of product defects. Although decentralised and autonomous decision-making and reliable real-time control are of paramount importance, conventional cloud computing naturally falls behind, owing to the centralised computing processing and the requirements for persistent connectivity. On the contrary, fog computing goes beyond and supports computation offloading of heavy tasks via autonomously and locally operated IIoT nodes, being close to the edge of the network, which in turns leads to decreased network traffic, improved scalability and efficiency, and enhanced security [30]. Recently, cloud/fog/edge architectures for data-intensive IoT applications, relying on efficient knowledge extraction from data existing in different areas of the decentralized hybrid clouds and within data lakes, in the form of unstructured data, were presented [31]. In the meantime, using ML at the edge can offer advanced prediction capabilities and efficient resource management given the resource-constrained nature of IoT-based devices. Next, recent research approaches based on ML technologies that promise to remedy the aforementioned challenges are described.

In Reference [32], an industrial CPS was described that employs fog computing and facilitates the delivering of real-time embedded ML applications through cyber-physical industrial interactions, while attaining security and privacy. More importantly, the cloud platform stored production-ready ML models in industrial operations, encoded as predictive modelling markup language (PMML) for various applications, which were divaricated and executed by the locally deployed fog nodes. Then, real-time predictions and decision-making, for example, control changes, could be provided without requiring persistent connection to the cloud. A three-layer architecture was proposed, where the sensing layer acquired real-time data measurements and incorporated a software agent to facilitate the communication between physical and cyber-environments. At the same time, the fog layer undertook the reception of inbound data streams and execution of PMML-encoded ML models, while the cloud layer was responsible for maintaining the metadata about each fog gateway in a global repository. It was noted that the PMML model was based on an existing SVM supervised learning model formally defined by a separating hyperplane, which is capable of predicting faulty heating operations by performing linear and non-linear classification and regression analysis in labeled training data. A comparison and performance assessment through multiple load/stress tests in industrial control processes between the underlined CPS and conventional cloud-based systems was performed, in terms of reliability and consistency. The results revealed the superior performance of AI-aided fog/cloud computing in several real-time decision-making and self-optimising engineering scenarios, where circumstantial failures may occur. Although the proposed architecture has significant benefits, it also has constraints mainly owing to the inadequate processing capabilities of fog nodes.

A reference four-layer cloud-assisted smart factory architecture was presented in Reference [33] that consisted of a smart device layer, a network layer, a cloud layer, and an application layer. By integrating these layers and applying AI techniques to each of them, a unified and coordinated smart factory environment can be realised that ensures flexibility, reliability, and efficiency. The smart device layer comprised basic smart devices in the production cycle, for example, robotic arms and automated guided vehicles (AGVs) and suggested the application of AI to machine vision and path planning applications. Besides, the network layer mainly included IWSNs, where the AI algorithms aim at improving the device-to-cloud (D2C) and device-to-device (D2D) communication and enhance the network resource distribution and load balancing. Moreover, exploiting AI in the cloud layer can effectively improve the processing of large-scale manufacturing data and resource management. Finally, adopting AI in the application layer resulted in dynamic preventive maintenance, resource reconstruction, and context-aware services. Nevertheless, technical challenges regarding the large amount of diverse and unstructured data, the system complexity and heterogeneity, the resources allocation, and the cloud security also exist and should be handled.

A CPS IoT-based architecture for manufacturing processes that supports multiple products, internal tasks and intermediate outsourcing flows was proposed in Reference [34]. To continuously improve the overall quality of the processes and dynamically control the mixed data flow issues from internal/outsourcing processes, a cloud computing control server was implemented, where the developed IoT beacons detected the status of the production machines and processes, collected sensor data, and wirelessly interacted with this server through the IEEE 802.11ac protocol at 5 GHz. Besides, the Intel IoT Analytics cloud module [35] was responsible for the data gathering task. To further strengthen the proposed architecture by introducing prediction capabilities, DL-based analytics were exploited to process the data and execute the fault detection classification. As indicative paradigms of the application of this architecture, the manufacturing processes for vehicles’ high intensity discharge headlight and cable modules were considered. More specifically, three learning algorithms were tested; the supervised learning support vector regression (SVR) that can perform real-value function estimation, the radial basis function (RBF) that can be used in various kernelised learning algorithms and in function approximation, and the deep belief learning-based DL (DBL-DL) that includes four layers, that is, one visible layer handling inputs from IoT sensors, two hidden layers extracting classification features and one output layer. Among them, the DBL-DL model was the most efficient for recognizing defect types. However, it is highly important to develop and apply accurate and fast analytics in the cloud layer along with big data techniques.

In an effort to accurately and in real-time detect the types and degrees of product defects in multiple assembly lines of industrial environments, a robust inspection system, named DeepIns, was implemented in Reference [36] aided by fog computing to offload the computation process to fog nodes and a DL-based CNN classification model. This model contained lower-level CNN layers to facilitate feature extraction during data uploading, as well as higher-level CNN layers to realized defect classification and degree regression. The proposed system was capable of processing big data with low response latency and network traffic and combined three modules; the fog-side and server-side computing modules and the back-end communication module. To investigate the performance of this system, image processing was realised using image filters, detecting surface defects by pixel chip pads. The data set included ten categories of defects, each of which contained 200 images for training and 50 images for network testing. The experimental results demonstrated the robustness and efficiency of the proposed method, regarding the recognition of defects against two well established methods, that is, the contour detection approach that uses contour classifiers to determine the category of defects and the pixel-based method that uses a classifier trained with pre-defined pixel-based features of input data. To further improve the efficiency of this system, multiple fog devices should be simultaneously used.

In Reference [37], a three-layer distributed ML model for an industrial monitoring system was presented that processes the data stream in the fog and intends to save spectrum and minimize the energy consumption by restraining unnecessary uplink data transmission. This model incorporated an IIoT cloud back-end storage point, implemented using the ThingsBoard platform, a fog middle layer, implemented using small resource-constrained Raspberry Pi devices, and a lower IWSN layer, consisting of TelosB motes with sensor devices and a gateway. The proposed model includes both learning and monitoring tasks in the sensor device, as well as simulation of the data streams in the fog node. The sensor nodes initially send information data to fog nodes, triggered by changes in the values of certain model parameters. The fog node simulates the data streams and recognizes local anomalies and faulty behavior of sensor devices, whereas the cloud gathers the local information from the fog node. To verify the performance of this model, a real-world test-bed was implemented with three different indicative noisy data streams as input, while a moving average model was used as a reference model for comparison purposes. According to the results, the proposed model reduced up to 98% the packet transmission attempts by sensor devices with minimal accuracy loss, whereas the end-to-end delay was constrained to 180 ms. By reducing its complexity, the proposed model can be used in devices with limited resources. Also, effectively prioritizing the packets can increase the reliability and flexibility of this model.

Since the wireless channels of IIoT are highly dynamic, typical techniques that enhance energy efficiency and extend the battery lifetime, for example, predictive transmission power control, cannot be directly applied. To overcome this issue, a forward centralised dynamic approach was proposed in Reference [38] that effectively manages the execution time and transmission power level of sensing, processing, and transmission processes of AI-driven IIoT devices at the edge. More importantly, FCDAA aims at adaptively optimizing the sleeping time of IIoT devices and the transmission power, while attaining enhanced reliability. A system-level battery model was also reported that inspects the duty-cycle and energy dissipation in these devices, along with a data reliability model over hybrid transmission power control and duty-cycle network associated with the wake-up and sleep periods of devices. To experimentally test the performance and reliability of the proposed method, an appropriate test-bed with IoT devices was implemented and industrial data sets were generated and processed. The Monte Carlo simulation results demonstrated the high reliability of this method in both static, that is, product processing and dynamic, that is, vibration and fault diagnosis, industrial applications, where the received signal strength indicator and the packet loss ratio were the key performance indicators. Although the integration of TPC and duty-cycle management mechanism for AI-based IIoT devices increases the complexity of the proposed FCDAA and the delays, this integration meaningfully decreases the energy consumption.

In Reference [39], a fog computing-based framework was described for remote sensing, monitoring of the health conditions of equipment, and fault detection in industrial environments. By enabling fog computing, this framework aims at attaining low network latency while ensuring reliability, scalability, and cost-effective storage of huge amounts of data. Also, this framework included an online process monitoring system, collecting real-time data, IWSNs, communication protocols, and ML-based predictive analytics. The competence of this framework was evaluated by conducting a real-world experiment on a factory floor that involved monitoring of the vibrations and energy consumption of pumps in a power plant and computer numerical control (CNC) machines. To gather real-time data, multiple wireless sensors, that is, current transducers and accelerometers, were installed on pumps and CNC machines. Each sensor was equipped with a ZigBee wireless module, in order to transmit the measured sample data sets to the private cloud, whereas sample data sets were sent to the Azure public cloud for data analysis. A supervised random forest (RandF) ML algorithm was used on the Amazon elastic compute cloud (EC2) for the development of a predictive model. This ensemble learning algorithm constitutes a simple and flexible method for classification and regression and consists of tree-structured classifiers. Based on this algorithm, multiple decision trees, which provide a classification for input data, are randomly created and merged into one “forest”. Then, the algorithm collects the relevant classifications and generates the prediction. The results underlined the accuracy of the predictive model, as well as the short training time associated with it.

Table 2 includes the targets of the various architectures and the ML solutions that they support in the context of Industry 4.0.

## 3. Fault Detection

In the context of Industry 4.0, fault detection and diagnosis is a crucial and demanding process due to the autonomous and self-optimised operation of machines and the wealth of data that is collected in real-time [40]. Currently, the use of diagnostic software for functional tests involves actual manufacturing data in real settings, providing poor diagnostic accuracy while technicians are required to perform several debugging rounds, as well as physical probing to identify the root cause of faults, thus demanding increased amounts of time for repairs, reaching several days or even weeks [41]. In ML-based approaches, the big data that is acquired by the monitoring system must be timely processed in order to correctly detect abnormal operation and faults. Fault detection and diagnosis mainly involves three steps, that is, data collection, data processing for feature extraction and finally, fault classification [42]. Relevant solutions for fault detection are mainly based on supervised, unsupervised and deep learning methods.

### 3.1. Supervised Learning-Based Solutions

In the area of supervised learning, several studies have employed ML methods to tackle issues of industrial fault detection and diagnosis. In Reference [41], the authors targeted the development of an automated diagnosis tool for circuit-boards, in order to reduce human effort and improve the diagnostic accuracy. The intelligent detection and diagnosis solution relied on three ML classification methods, that is, ANNs, SVMs and weighted-majority voting (WMV), where the latter combined the benefits of ANNs and SVMs. These methods were trained using repair history data while, fault instances stemming from failure logs and subsequent repair actions were exploited to train the classification models. The comparison in terms of accuracy and resolution among the three ML methods and conventional diagnostic software showed that WMV provided the best performance, offering optimal repair guidance. Moreover, the proposed ML-based methods represent significant improvements compared to conventional manual fault detection and diagnostic software. From the performance evaluation, when three attempts are performed, according to the proposed optimal repair suggestion set, the accuracy of the conventional diagnostic software falls below 50% while the adoption of the ML-based diagnostic system allows more faulty boards to be successfully repaired, reaching up to 77.5% in low volume manufacturing and 98.7% in high volume. Nonetheless, in Reference [43], it was shown that the accuracy of these methods may be compromised in cases where repair logs are fragmented, resulting in missing errors, or syndromes during the diagnosis process. Thus, as missing syndromes might lead to erroneous repair guidance, the authors presented a board-level fault diagnosis system to mitigate their effect in different ML models. In greater detail, the log files containing the syndromes from a faulty-board were analysed and preprocessed prior to root-cause analysis. The ML methods included SVM, ANN, Naive Bayes, and Decision Tree and were implemented on WEKA [44], using training data from two synthetic boards and two industrial boards. The extensive performance evaluation showed that the fault diagnosis performance significantly varies, depending on two factors. Firstly, different ML algorithms perform better under different missing-syndrome handling methods, for example, ANN is superior when label imputation is adopted, while its performance deteriorates under the decision-tree model. Secondly, the fault diagnosis systems may set different goals, including not only high fault detection and diagnosis accuracy, but also the reduction of irrelevant syndromes.

Another work investigated the impact of imbalanced data sets on the performance of SVM binary classifiers [45]. In these cases, employing sampling methods like the synthetic minority oversampling technique (SMOTE) can balance the data set prior to training by a classifier. Still, in nonlinear problems, SMOTE experiences difficulties as minority instances in the feature space of the classifier, instead of the input data space are generated. In order to overcome this limitation, weighted kernel-based SMOTE (WK-SMOTE) was presented, performing oversampling in the SVM classifier’s feature space. WK-SMOTE was shown to improve the performance of detecting the stage of degradation in the insulation of high-voltage electrical machines in comparison to other methods, according to multiple trials of imbalanced data sets. More specifically, when WK-SMOTE is applied on this multi-class imbalanced classification problem, the highest G-Mean score, equal to 0.371 is achieved, as well as the best accuracy equal to 88.1%, among the compared algorithms. Similarly, supervised ML for fault detection with imbalanced data and concept drifts, that is, changes in fault patterns, due to machinery aging or after repair/replacement was examined in Reference [46]. For this purpose, ensemble learning was adopted, integrating several offline classifiers and a three-stage method was developed to detect abnormal machine operation in a smart factory. During the first stage, an ensemble classifier was trained using an improved dynamic AdaBoost.NC classifier and the SMOTE method was adopted to reduce the effect of data imbalance. Then, in the second stage concept drifts in imbalanced data were detected, employing a linear four rates method. Finally, the third stage created a new ensemble, by relying on AdaBoost.NC and SMOTE. The experiments that were conducted with imbalanced data sets, revealed that the proposed method can successfully detect abnormal operation with an accuracy rate in minority-class data of over 94%. Detailed comparisons between ML methods were presented in Reference [47] for fault detection in semiconductor manufacturing with imbalanced data. In greater detail, three sampling-based, four ensemble, four instance-based algorithms, and two SVM methods were evaluated, in two cases, with each one comprising 50 data sets. In the first case, etching process data was acquired while the second case was based on chemical vapor deposition process data. From the trials, it was shown that the instance-based algorithms exhibited the best performance, in terms of G-mean, F-measure, and area under the curve, even when the imbalance ratio increased. Thus, for settings where wafer defects stem from different process parameters, while the available training samples lie in a small subregion of the space form from these parameters, defective patterns can be only learned within this subregion. So, efficient model training necessitates the availability of defective data from the majority of the defective patterns, even if they occur with low frequency. Still, instance-based fault detection addresses such issues, surpassing the unavailability of defective samples.

Next, the development of a monitoring system, leveraging the availability of big data from an automotive manufacturing plant was the subject of Reference [48]. As real-time monitoring is necessary to avoid sudden interruptions in the production process, IoT sensors collecting a wide range of data, such as temperature, humidity and measurements from accelerometers and gyroscopes were used. Fault prediction was based on a hybrid prediction scheme, consisting of density-based spatial clustering of applications with noise (DBSCAN) to detect outliers and RandF classification to remove outliers prior to fault detection. Furthermore, this hybrid scheme was tested at a real automotive manufacturing plant and results suggested that hybrid DBSCAN-RF can offer improved accuracy, compared to classification methods, such as Naive Bayes, RandF and multilayer perceptron, among others. In greater detail, DBSCAN achieved an accuracy of 100%, compared to other classification models, while DBSCAN-based outlier detection with RandF improved the accuracy of standalone RandF by 1.462%. At the same time, the integration of DBSCAN with Naive Bayes, logistic regression, and multilayer perceptron improved the performance of their conventional versions by 3.173%, 0.567%, and 2.026%, respectively.

### 3.2. Unsupervised Learning-Based Solutions

Regarding unsupervised learning methods, there have been various works presenting fault detection solutions. The authors of Reference [49] aimed to alleviate the burden of manual fault information extraction, relying on previous knowledge and diagnostic skills, by developing an intelligent fault detection scheme. So, a two-stage unsupervised learning method using a neural network (NN) was employed, where in the first step, sparse filtering for feature extraction from the mechanical vibration signals was performed. After, the second step was based on softmax regression for automatic classification of the machines’ health conditions. The proposed method was validated in two scenarios, that is, fault detection for motor bearing and locomotive bearing, showing that, as the volume of unlabeled data increased, the accuracy performance was improved. The proposed two-stage unsupervised learning with an NN improved the accuracy of fault diagnosis as number of the unlabeled data increased. Furthermore, the weight vectors of sparse filtering exhibited similar properties to Gabor filters, serving as excellent band-pass signal filters, providing an insight on the way that unsupervised feature learning deals with mechanical signals.

Then, for renewable energy systems, based on wind turbines, the work in Reference [50] introduced an autoencoder and explored its application on fault classification from collected raw vibration signals, integrating both supervised and unsupervised learning methods. More specifically, a fault diagnosis scheme consisting of multiple hierarchical extreme learning machines (ELM) was presented, concatenating a forwarding list of ELM layers, with each one performing independent data processing. ELM represents a learning method that is suitable for multi-class classification, due to its multi-input structure and single-hidden feed-forward networks, achieving better performance compared to SVM [51]. ELM performs representational learning towards data preprocessing, feature extraction and dimension reduction. During data preprocessing, the ELM-based autoencoder was used for data representational learning, providing the feature reconstruction and in the next step, the ELM network extracted a compressed low-dimensional representation. The ELM-based autoencoder increased the classification accuracy by 5–10% when compared with other popular classifiers, such as SVM and relevance vector machine (RVM), searching the optimal solution from the constructed feature space. So, it was revealed that accuracy and efficiency of multiple fault detection for wind turbines can be enhanced, while this solution can be scaled to other industrial settings.

Unsupervised learning solutions for CPS have been developed in Reference [52]. Focusing on monitoring the machine spindle during its operation in a CPS, multidimensional classification using ML clustering was developed. The main target of the clustering algorithms was to partition the collected data set into clusters, by evaluating metrics, such as Euclidean distance or probability distributions, that do not consider the physical interpretation of each variable, thus resulting in uncoupled knowledge discovery. In addition, a performance comparison was conducted for the three clustering algorithms that were employed, that is, K-means, hierarchical agglomerative and Gaussian mixture models, evaluating their accuracy to spindle performance knowledge during high load operation. It was concluded that, although the Gaussian mixture model incurred higher implementation complexity, it was capable of detecting clusters that were more relevant to the operating conditions. Additionally, the agglomerative algorithm provided a detailed analysis of the cycle’s but it was severely influenced by inherent variables, such as angular speed, leading to possible interference during knowledge discovery. Finally, K-means clustering performed similarly to the hierarchical agglomerative algorithm but it was adequately faster, thus enabling rapid data set analysis.

### 3.3. Deep Learning-Based Solutions

In order to improve the performance of fault detection and overcome the limitations of conventional ML methods, DL-based solutions have been developed. DL relies on raw data and multiple layers, with each one designed to be simple and non-linear, being able to automatically create new representations of the data and provide accurate detection or classification [53].

In industrial applications, where fault detection uses noisy mechanical data, a DL solution named LiftingNet was presented in Reference [54]. LiftingNet can be adaptively trained using mechanical data sets and perform classification without pre-defined features. For validation purposes, two motor bearing data sets were used, that is, the Case Western Reserve University (CWRU) and a motor bearing data set with varying rotating speed. It was shown that LiftingNet was able to adapt to different tasks and provide fault classification, even using only noisy mechanical data, surpassing the performance of conventional SVM classifiers, in cases where the desired data is usually difficult to trace and process. Still, LiftingNet was characterised by three limitations. Firstly, fault severity could not be assessed, as the amplitude of mechanical data was normalized in order to feed the various rotating speed signals into the network. Secondly, fast training and overfitting prevention techniques were not applied, thus limiting its performance. Thirdly, better designs for the loss function and the final classifier should be integrated, towards improving the behavior of LiftingNet. Another paper examined fault pattern and crack size identification by exploiting mechanical data and more specifically, vibration acceleration signals from machine bearings [55]. Thus, two-layered bearing fault detection was developed, relying on a hybrid feature pool extracting information from the raw vibration signals, addressing their non-stationary behavior, due to different crack sizes. Then, sparse stacked autoencoder (SAE)-based DNNs were employed for data classification. From the results, it was highlighted that the proposed two-layered bearing fault detection method improved the detection accuracy, compared to SVMs and back-propagation NNs (BPNNs) alternatives, for varying fault severity, independently of the nonlinearities in the vibration signals. Still, for roller fault identification, its performance was slightly degraded, underpinning the need for improved signal processing algorithms.

The issue of handling changes in the signal features and performing early fault detection was discussed in Reference [56]. Due to these variations, it is challenging to decide whether or not, they stem from abnormal mechanical faults or scheduled changes during machine operation. Aiming to tackle this problem, a three-step early fault detection method, applicable in time-varying workloads was given in detail. In the first step, DL was adopted for the automatic selection of the impulse responses from long-term mechanical vibrations. Next in the second step, an algorithm was developed, extracting the properties of machine tools from the selected impulse responses. Then, the third step involved an indicator based on these properties, being used for health status prediction of the machinery. The DL-based solution was evaluated, using a large volume of data during a period of 228 working days in an automobile factory. It was shown that the reliance on the extracted dynamic properties offers significant improvement in health prediction, compared to conventional signal-based features, such as time-domain features, frequency-domain features and wavelet transform.

The detection of failures in the gearbox of mechanical equipment, and more specifically wear, pitting and broken teeth that may cause interruptions and workers’ injuries, was the topic in Reference [57]. Since current data-driven solutions have limitations due to using specific data classes, it is necessary to study various data characteristics to improve the detection accuracy. As a result, the developed DL-based solution performs data acquisition of mechanical vibration signals, deriving representative features. Then, an NN for fault detection was constructed, based on the multi-layer gated recurrent unit (MGRU) model. MGRU provides feature representation with stronger similarity to the original data. So, each neuron layer, sequentially learned more abstract representations and offered better classification results. Regarding the accuracy performance, MGRU improved the accuracy of gear fault detection without incurring excessive computational complexity, when compared to the long short-term memory (LSTM), the multi-layer LSTM (MLSTM) and the gated recurrent unit (GRU) model. Then, the separability analysis showed that MGRU was able to extract the information from the plethora of mechanical signals and efficiently identify the different fault types. Moreover, MGRU exhibited strong robustness against the variations of parameters in the experiments, maintaining a stable classification accuracy. Further advancements to DL for fault detection, due to machine bearing, were reported in Reference [58] where a snapshot ensemble CNN (SECNN) was proposed. Snapshot ensemble learning based on the cyclic learning rate (CLR) scheduler and the combination of local minima has the potential to improve fault detection performance but requires experience-based pre-defined CLR information. In order to automatically determine the appropriate learning rate for SECNN under different data sets, a three-step approach was given. Initially, max-min cosine cyclic learning rate scheduler (MMCCLR) was designed to mitigate the effect of other parameters on the learning rate. In the next step, a new learning rate testing, namely logLR Test, was employed for proper learning rate range estimation of the MMCCLR. The proposed SECNN with MMCCLR was experimentally tested using the bearing data set of CWRU, the self-priming centrifugal pump data set, and the bearing data set of the machinery failure prevention technology. From the results, it was concluded that SECNN with MMCCLR surpassed other baseline methods, based on stochastic gradient descent (SGD), including SGD with decay ratio (SGD-Decay) and stepwise decay (Step-Decay). However, some limitations were highlighted, regarding SECNN. More specifically, it was observed that SECNN did not consider imbalances among normal and fault data. At the same time, pre-defined fault types should be often used for training, thus resulting in incorrect classification of faults that did not exist in the training data set.

In a different setting, fault detection for computer-based assembly lines of the automotive industry was studied in Reference [59]. This process is usually performed through boundary checking while the analysis of complex non-linear signals is manually performed by experienced personnel. Thus, a DL-based automated fault detection and isolation (FDI) method was presented, using the individual signals from various software and hardware sources of the assembly line. After, data encoding took place and signal values corresponding to categorical entities were encoded through one-hot-encoding. At the same time, for continuous signals, a specific range was considered and divided into a fixed number of bins, based on the statistical properties of the signal. In order to detect correlations among different inputs, the spatial transformation of the input space into a vector-space embedding was performed through a set of deep auto-encoders (DAEs). As the continuous space of vector embedding requires further processing in order to correspond to specific system states, hierarchical clustering processed the extracted features from the DAE. From the performance evaluation, it was demonstrated that the proposed DL-based FDI was capable of processing and modelling the large volume of multi-type spatial-temporal manufacturing data, at a high accuracy, in real-time working conditions, surpassing alternative methods, including template-based, rule-based and Bayesian-based FDI.

Table 3 summarizes the industrial settings and the ML solutions that were adopted for fault detection and diagnosis in Industry 4.0.

## 4. Predictive Maintenance

Another important process that can greatly benefit from the application of ML, is predictive maintenance. More specifically, the availability of a wealth of data, stemming from the various processes and machine state information enables the timely and accurate prediction of *when* machinery requires maintenance, as well as *how* maintenance operation can be improved. Even when clear indications of machinery wear is not available, predictive maintenance leverages on data acquired during production to identify the characteristics of this degradation. In this way, costs and downtime duration can be reduced while increasing the production output. The application of BDA and data fusion for predictive maintenance purposes has been recently investigated within the contexts of semiconductor manufacturing and smart factories, in References [60,61], respectively.

### 4.1. Supervised Learning-Based Solutions

The first category of ML-based solutions for predictive maintenance relies on supervised learning and comprises the majority of relevant works. In Reference [62], multiple classifiers (MC) were adopted for predictive maintenance, regarding integral type faults in semiconductor manufacturing, that is, failures occurring due to the accumulative high usage and load on the machinery parts. Thus, the developed method aimed at addressing the issue of imbalanced data sets, usually existing in maintenance classification problems [63] and enabled maintenance under statistical cost minimization. Towards this end, MC components operating with varying prediction horizons, improved performance targets, such as predicting frequency of unexpected breaks and unexploited lifetime, imposing maintenance decision rules in a dynamic manner and tackling high-dimensional data problems. The MC supervised ML method was implemented in a semiconductor manufacturing maintenance process, revealing that better performance can be achieved, compared to conventional preventive maintenance solutions and predictive maintenance alternatives, relying on single SVM and k-NN classifiers. Thus, the MC method was capable of providing the lowest operating costs while being robust against varying values of parameters, determining the frequency of unexpected breaks and the amount of unexploited lifetime. Another work investigated the remaining lifetime detection of key machinery components for predictive maintenance purposes by analyzing heterogeneous data that were collected through multiple sources [64]. More specifically, an ML-based solution was developed for structuring such data sets, considering the spatiotemporal property, and invisible factor modeling for reduced downtime and energy-efficient machine operation. The adopted systematic procedure comprised structuring of industrial big data, and semantic web technology for semi-structured data, as well as target recognition, detection and tracking for unstructured data. Then, multi-scale analysis was performed for structured data characterisation, identifying hidden patterns, while spatial envelope analysis extracted independent subsystems and time-frequency analysis performed signal decomposition. The performance assessment of the multi-scale analysis method showed that it improved the accuracy of remaining lifetime prediction, compared to an alternative ANN method using a single source of information. More importantly, it was concluded that exploiting heterogeneous data from multiple sources enables novel methods of predictive maintenance, task scheduling and machining process optimization towards reducing the energy consumption.

Next, predictive maintenance based on heterogeneous data, such as cutting forces, vibration signals and acoustic emissions, from milling machinery was the main focus in Reference [65]. The authors adopted ensemble learning RandF where each individual decision tree corresponds to a regression tree, as tool wear represents the gradual failures in the operation of cutting machinery. Also, the bagging, slipping and stopping criteria for the ML-based solutions were given and comparisons with ANNs with a single hidden layer and SVMs alternatives were presented, using a data set collected from 315 milling trials. It was observed that although RandF required longer training than ANNs and SVMs, it provided the highest accuracy in predicting tool wear. An SVM solution was proposed to address issues in shop-floors where the main production is performed, through automated machines, workers or both, in Industry 4.0 environments, millions of devices and sensors can produce a wide range of data for predictive maintenance. So, in Reference [66], ML based on SVM was presented, modelling the conveyor belt system through the M/D/1 queue. In this setting, SVM predicted instances of abnormal operation where machine overloading or slow downs occurred, by detecting changes in the queue parameters. For any abnormality, the situation was solved by reconfiguring the manufacturing system. This enabled a flexible system, even if an abnormal situation occurred, as illustrated through simulations where automated detection of abnormalities and self-healing were observed.

As small and medium-sized enterprises (SMEs) constitute a major group within the Industry 4.0, providing specific benefits in terms of innovation and willingness to adopt novel solutions, as well as limitations regarding their workforce and economic resources, the work in Reference [67] proposed a low-complexity solution for predictive maintenance, aimed at SMEs. Currently, existing ML-based methods rely on complex, algorithms, such as linear-discriminate analysis (LDA), decision trees and neuro-fuzzy networks. Moreover, such methods are based on significant amounts of data being collected for classification and feature extraction. For these reasons, the authors proposed a low-complexity algorithm for online feature dimensionality reduction and automated machine status detection and prediction through an NN. The proposed solution included two steps where in the first, add-on sensors collected machine status data. Then, data parsing and feature extraction were performed, prior to dimensionality reduction and feeding of the results, as input into NNs for training and prediction model creation. For verification and validation purposes, the NN-based method was implemented using real machine data from a spring manufacturing factory, employing add-on triaxial accelerometers for data collection. Comparisons with other methods, such as LDA, showed that highly attractive trade-offs between accuracy and prediction can be attained, thus facilitating the adoption of ML-based predictive maintenance by SMEs. More specifically, the proposed method achieved an overall accuracy of 93.1%, equal to LDA, but with the capability of being implemented with more simple hardware, thus avoiding the need for expensive and specialised hardware.

In cases where machine conditions dynamically vary in time due to concept drifting with aging, failures and repairs, predictive maintenance is a challenging process. More specifically, patterns stemming from the operational data could significantly change, thus resulting in reduced prediction accuracy. In order to tackle this issue, especially when only a single classifier type is used, ensemble learning based on MC types and diversity was employed in Reference [68]. The basis for the proposed solution is diversity for dealing with drift (DDD) [69], enabling data diversity manipulation for constructing ensemble models, with each one comprising several base classifiers to address concept drifts. However, the multiple classifier type DDD that was developed, consisted of multiple classifier types, dynamic weight adjusting, and data-driven adaptation to concept drifts for offline learning. In this way, timely adaptation to concept drifts and high prediction accuracy can be harvested. Moreover, to relax the computational requirements, the distributed cloud computing framework, called MapReduce was adopted [70]. From the simulations that were conducted with a concept drift data set, the efficiency of the multiple classifier type DDD was outlined, adapting to concept drifts by appropriately updating the classifier weights, leading to an accuracy of 87.99%.

### 4.2. Unsupervised Learning-Based Solutions

Currently, there exist few works presenting unsupervised learning solutions for predictive maintenance. In Reference [71], a complex system is given, performing IoT data collection, storage, processing and visualization, prior to predictive modelling. This system provides structured and unstructured data ingestion from multiple sources, as well as data management via data lakes, based on file and data base management systems, such as Hadoop and Apache Hive, among others. The joint consideration of big data and cloud computing, during the predictive stage encompasses data quality processing and MapReduce-based distributed principal component analysis (DPCA). DPCA exploits unsupervised learning for training, using unlabeled data which is necessary in large-scale manufacturing plants, characterised by heterogeneous data. The system was subject to long-term evaluation in a real industrial environment and compared learning algorithms based on K-means, square predicted error (SPE) and DPCA-based T-squared. It was observed that SPE exhibited a more stable behavior, in terms of fault detection, while K-means and T-squared were more sensitive in instances of multi-variable value changes. Independently of the adopted ML algorithm, the system enabled real-time notification of abnormal operation, even several days prior to an actual failure. Next, the work in Reference [72] aimed at integrating real-time and historical analysis, towards self-optimization for predictive maintenance. Thus, an intelligent data analysis and real-time supervision (IDARTS) framework was presented for data collection and creation of context-aware data analysis and evaluation. IDARTS was designed according to the plug-and-produce functionality, supporting predictive maintenance with dynamic system virtualization, addressing changes at the shop-floor level. The data output from the virtual resources was used to train a K-means clustering classifier. Moreover, context-awareness and HMI allowed the system to either adopt the operation parameters towards self-reconfiguration, or guidance to an operator for safeguarding normal production conditions and product quality. From the experiments, it was revealed that IDARTS provided scalability and adaptability to production changes, in terms of shop-floor layout while performing data analysis in runtime environment, without needing additional programming, down time or redeployment.

### 4.3. Deep Learning-Based Solutions

DL has emerged as a significant advancement in data-driven analysis and optimization for industrial applications, especially for those focusing on the accurate prediction of RUL. The authors of Reference [73] presented a device electrocardiogram (DECG) framework, employing a deep denoising autoencoder (DDA) and regression operation to enhance the accuracy of RUL prediction of industrial machines. DECG avoids sensor installation and acquires temporal data for each operation at the device or operator level, from the programmable logic controller (PLC). Also, DECG overcomes the necessity of experts in complicated production issues, greatly reducing maintenance costs. More specifically, DECG collected data relative to the machines’ cycle time and through DL and a wide range of run-to-failure data, achieved automated feature extraction and accurate prediction. The proposed solution was put against a conventional factory information system and results illustrated that DECG was able to identify the behaviour of operation working time and offered more fine-grained training data, facilitating DL in accurately predicting RUL. Thus, the proposed solution can lead to reduced DL overfitting and improved RUL prediction accuracy. Next, RUL through deep transfer learning (DTL) was investigated in Reference [74]. DTL addressed feature transfer in DL networks and relied on three strategies, that is, weight transfer, feature transfer and weight update. Thus, DTL with feature transfer learned the joint features among multiple objects, improving the prediction accuracy of RUL, thus showing the advantages of DTL. In greater detail, initially, a SAE network is trained off-line with historical run-to-failure data containing RUL information of a cutting tool. Next, the trained network is transferred to an operating tool towards performing on-line RUL prediction. In this way, RUL prediction was performed without interrupting the machine tool’s operation. Also, efficient RUL prediction was achieved without requiring excessive historical failure data sets. As current RUL prediction methods experience difficulties in extracting heterogeneous features from high-dimensional massive signals, the predictive maintenance performance is threatened. In Reference [75], data-driven machine health monitoring was developed, employing adaptive kernel spectral clustering (AKSC), combined with deep long short-term memory recurrent NNs (LSTM-RNN). In greater detail, a three-step approach was adopted, first including, frequency and time-frequency domain extraction from massive signals and an Euclidean distance-based algorithm for identifying the machine degradation features. Then, AKSC was presented, for detecting anomalies in the machine operation, based on multiple degradation features. In the last step, LSTM-RNN was created for updating and predicting the machine failure time. The performance of AKSC with LSTM-RNN was evaluated, using a set of test-to-failure experimental data, outlining its superiority compared to other methods, in terms of average error, root mean square error and accuracy. The improved performance of LSTM-RNN was attibuted to its ability of extracting the long-term spatiotemporal dependency of different degradation parameters, towards provding short-term failure prognostics. Furthermore, predictive maintenance in ultra-precision manufacturing applications has been studied in Reference [76]. So, a DL data-driven framework was proposed, fusing multiple stacked SAEs to perform tool condition monitoring. The DL-based solutions consisted of a training model that was able to process multiple parallel feature spaces from time-, frequency- and wavelet-domains data, extracting low-level features and a feature fusion structure for learning higher-level features relevant to tool wear. In addition, feature extraction and classification performance was improved by a modified loss function. A data set representing a real manufacturing process was used to evaluate the performance of the DL-based solution. It was shown that by exploiting the feature learning ability of deep layer models and the heterogeneous feature spaces, the proposed solution successfully classified the tool wear condition with over 96% accuracy, outperforming BPNN and SVM alternatives.

Table 4 includes the specific industrial environments and the respective ML solutions that were employed for predictive maintenance of equipment in Industry 4.0.

## 5. Security and Threat Detection

Industry 4.0 invokes a wide variety of sensors and actuators, IT systems with fog/cloud data-driven processing and BDA, as well as wireless and wired network components, along with different standards and specifications. These key elements, coupled with the end-to-end digitization have the potential to offer many advantages and significantly transform the supply chain and the product lifecycle of the manufacturing system and make them more efficient, decentralized, and sufficiently-connected. However, Industry 4.0 also bring various security challenges and caveats, due to the integration of new technologies and architectures, thus making the industrial infrastructures more susceptible to new forms of attacks, such as product tampering, service interruption, infiltration, intellectual property loss, and spear phishing attacks. More importantly, these attacks not only trigger malfunction, but also leak important information. For instance, cyber-attacks, such as Stuxnet computer worm attacks [77] tend to increase, along with the increasing levels of connectivity. The heterogeneous, diverse, and large-scale nature of the aforementioned enabling technologies and the complex physical industrial environment itself hinder the application of traditional information technology security countermeasures, for example, firewalls, and the detection of potential security vulnerabilities.

Various challenges arise for maintaining the security requirements for integrity, confidentiality, and availability by protecting each subsystem from unauthorized access, use, disclosure, disruption, improper modification or destruction of data [78]. More specifically, integrity is related with consistency, accuracy, and reliability of both the information data and the physical components of the entire supply chain and the product lifecycle. Besides, confidentiality involves privacy issues in the horizontal and the vertical value chain of the industrial systems and preventive maintenance data security, whereas availability includes resiliency and recovery of a typical distributed Industry 4.0 architecture.

Heretofore, the aforementioned issues have been highlighted in the literature [78], while several solutions, including cyber and physical detection methods, as well as standards (e.g., Reference Architecture Model Industry 4.0 (RAMI 4.0) [79], Industrial Internet Reference Architecture (IIRA) [80]) to safeguard manufacturing have been provided to realize an Industry 4.0 environment secure from known risks, vigilant against new threats, and resilient against zero-day attacks. Nevertheless, security issues in specific domains can drastically influence the operation of the entire system in a non-trivial and non-evident manner. As the cyber-risk to the next-generation industrial systems is estimated to increase, protecting the corresponding implementations against conventional and sophisticated cyber-attacks at all levels of the industrial processes represents a challenging task. To improve threat detection, ML is the key technology that enables fundamentally effective decision-making capabilities. By integrating cyber-security and physical data, ML not only facilitates the detection of cyber-physical attacks, but also, effectively enhances the accuracy and shortens the response time of security mechanisms. Although ML has been intensively applied both in physical security data and manufacturing systems, it has not been extensively exploited in manufacturing security. Physical security training data for ML can be acquired through voice recognition, fingerprint authentication, gait authentication, keystroke and other biometrics. Also, ML implementations in manufacturing involve real-time vision system for surface defect detection, weld defect defection, surface defect detection, preventative maintenance, and supply chain optimization.

To obtain accurate knowledge about cyber-threats and effectively recognize future malicious events in CPS, where multivariate time series data is dynamically generated, exploiting threat intelligence is of paramount importance. More specifically, threat intelligence enables efficient event and network traffic monitoring and analysis, using a signature-based method exploiting a predefined blacklist of existing attack signatures, an anomaly-based method taking advantage of profiles of normal events to recognize attacks or a hybrid technique, where an anomaly generally defines something that visibly deviates from the norm/standard and can be identified by analysing the sensed data or traffic patterns. In Reference [81], a novel threat intelligence scheme based on beta mixture-hidden Markov models (MHMMs) was proposed that intends to model the dynamic interactions of Industry 4.0 subsystems and discover known and unknown attacks, while surpassing existing signature- and anomaly-based methods. In this scheme, Industry 4.0 key elements, that is, CPS and IoT, interact and two principal components are included; a smart management module, handling the heterogeneous data sources of sensors, actuators and network nodes and a threat intelligence module monitoring and indicating abnormal activities and cyber-attacks in the physical and network domains. The latter is based on a Beta Mixture and hidden Markov mechanism (HMM), aiming at accurately and effectively detecting and discriminating normal and attack data. In HMM, the first component, namely the beta mixture model (BMM) [82] fits multivariate time series of physical and network data and stands for the input of the second component, that is, the HMM [83], which aims at estimating the posterior probabilities and identifying the latent structures for detecting indistinguishable normal and abnormal states. The performance of the HMM was enhanced by excluding irrelevant features and reducing sensor and network dimensionality, through an independent component analysis (ICA) technique [84]. Since publicly accessible real-world Industry 4.0 data sets are not available, the evaluation of the threat intelligence scheme was realised by combining two well-known data sets; the CPS data set of sensors and physical devices [85] and the UNSW-NB15 data set of network traffic [86]. According to the results, the proposed method surpassed other previously proposed anomaly detection methods, that is, methods that only create profiles from normal activities to differentiate attacks without defining the attack types, in terms of detection rates, false positive rates, and processing times. Although, the MHMM stands for a strong potential candidate for the monitoring and detection of both known and zero-day attacks, it requires a significantly large number of data samples for parameter estimation.

An ML approach to physical data was proposed in Reference [87] for cyber-physical attack detection. Two examples of attacks were considered and simulations, along with experimental demonstrations were carried out to verify the effectiveness of the proposed method on cyber- manufacturing system (CMS) security. In particular, ML was initially used to recognize malicious defects in three-dimensional (3-D) printing (additive manufacturing process) and so, 3887 simulation images with 512 × 512 pixels size were captured. Also, ML was used to detect two attack modes, that is, attack on design and attack on operation, in CNC milling process (subtractive manufacturing process) with acoustic signal data. In particular, the signal used in the simulation process was a time-serial amplitude numbers, created by a summation of sine-functions with fundamental frequency, harmonic frequencies and a Gaussian noise. Three ML algorithms were implemented with image classification; the supervised KNN and random forest learning methods that perform discriminant analysis and multi-way classification, respectively, and the unsupervised anomaly detection method that is capable of recognizing abnormal behavior and new types of attacks contrary to the supervised methods. Notwithstanding, the anomaly detection method necessitates the definition of particular rules for normal network behavior, which is not a straightforward process. The results indicated that the anomaly detection algorithm was capable of obtaining up to 96.1% accuracy in detecting malicious cyber-physical attacks in the 3-D printing, whereas RandF reached a mean 91.1% accuracy in detecting cyber-physical attacks in CNC milling process. Besides, the KNN method fell behind in terms of accuracy.

In Reference [88], a cost-effective context-aware DNN-based intrusion detection method was presented. This solution consisted of several hidden layers between the input and output layers and it was applied in smart factories, threatened by a plethora of advanced cyber-security threats. This unsupervised DNN-based learning method included hidden layers between input layer and output layer and involved three phases; data capture and parsing, odel build and inference, and threat visualization. More specifically, a multi-variety production system with heterogeneous facilities was considered, with interconnected devices and objects. Based on the results, this method can effectively detect anomaly signs and significantly reduce the risks of cyber-attacks. Specifically, the results underlined that by adopting the propose solution, the cyber-security performance metrics were improved up to 33%, compared to other conventional methods. However, the major drawback of this study is the limited data, which in turns hinders the generalization of the obtained results in other application domains.

In Reference [89], the security risks in industrial control systems (ICSs) were mitigated through a process-aware supervised learning data-driven defense method that detected various categories of attacks and distinguish between disturbances and malicious behavior. In particular, a robust non-linear supervised SVM model was developed that was capable of detecting abnormalities and malicious activities in real-time, distinguishing the attacks from process disturbances, while providing redundancy-based mitigation of detected attacks via automated control switching. A primary SVM along with separate SVMs were adequately trained using large data sets of normal operation and attack condition generated by multiple sensors of a complex, non-linear Tennessee Eastman (TE) process, in order to identify on-going attacks and discrimate their types, respectively. It is noted that the TE process stands for a well-defined simulation of a real chemical process that has been extensively used for comparative assessment of several data-driven fault detection and diagnosis methodologies in process control research [90]. To demonstrate a complete payload delivery mechanism and investigate the influence of process-aware attacks on the entire process, a hardware-in-the-loop (HITL) test-bed was exploited, which used realistic disturbances in a simulation model. The threat model was identical to that of the Stuxnet worm [77] and three attacks were assumed, that is, sensor, controller, and actuator attacks. These attacks aimed at modifying or spoofing data values and parameters of the process. Based on the experimental results, the proposed ML approach satisfactorily and accurately detected all previously unseen tested payloads with small delays, whereas false alarms were not observed contrary to conventional attack detectors that rely on maximum and minimum thresholds.

Table 5 lists the cyber-security targets of different relevant studies and the adopted ML solutions, guaranteeing the cyber-security in various Industry 4.0 settings.

## 6. Human–Machine Interaction

Although new requirements for digital technological advancements are emerging within next-generation complex industrial environments, the human factor remains critical, as traditional human-physical systems (HPSs) suggested, and stands for an essential component in attaining flexibility and adaptability in industrial processes. However, in technology-rich working environments, work division between human workers and machines is usually observed and the assigned tasks are categorised into human-specific and machine-specific. Contrary to the principle of the computer-integrated manufacturing (CIM), Industry 4.0 does not envision workerless production facilities, but rather pursues the synergetic integration of humans and machines in a shared working space, in the sense of a human-cyber-physical system (HCPS) [91], where the individual skills and talents of humans can be successfully exploited. Industry 4.0 imposes transitions on work division between human and machines, since humans are assisted by intelligent devices and machines, that is, human–machine cooperation, while interacting and exchanging information with the machines, that is, human–machine crossover collaboration. Therefore, there exists a need for a symbiotic environment that focuses on the effective collaboration and cooperation between humans and machines for handling combined task elements, while communicating over the IIoT [92]. Through this paradigm, higher product variability and customization, as well as increased production efficiency and quality can be ensured. Furthermore, humans excel at making decisions and exploring new solutions, under situations of uncertainty and incomplete information data. Also, humans demonstrate adaptive capabilities and can more efficiently propose useful policies, in a timely manner. Besides, machines can be used for rapidly and repeatedly storing, retrieving, and processing data, as well as for systematic pattern recognition. Next, we initially survey recent theoretical studies on HMI in Industry 4.0 and then, we underline works that simulated and implemented ML-based HMI solutions.

### 6.1. Theoretical Frameworks

To further improve the accuracy and the quality of industrial processes, the Intelligent Factory Space (IFS), an AI-inspired architecture that facilitates the interaction between humans and robots, was presented in Reference [93], emphasising on the safety and trust aspects, during the two-way communication between humans and robots. Also, the notion of patented intelligence (Pi)–Mind was described in detail in Reference [94], as an Intelligence-as-a-Service (IaaS) provider that transparently and proactively introduces human expertise on decision-making based on self-awareness. As the dynamic industrial CPS usually encompasses unexpected or emergency situations, conventional ML and predictive methods might not lead to the desired outcome. In this direction, Pi-Mind suggests that the experienced humans and AI-driven machines are an indivisible compound and share the responsibility for the impact of the decisions made in critical cases. This technology surpasses the rational decision-making by comprising models, techniques, and tools that capture and clone, that is, digital twinning, the creative cognitive human capabilities and the created Pi-Mind agents. Additionally, the concept of Industry 5.0 was introduced in Reference [95], which goes beyond automation and envisions the synergy between humans and autonomous machines to enhance creativity and efficiency. In Industry 5.0, two types of learners are observed; human operator as a learner and machine, robot, or computer as a learner.

As the human behavior is dynamic, an accurate characterisation or modeling of the human performance through conventional methods is not usually feasible. In general, humans are often considered, as a physical resource that can be characterised by statistical terms. As shown in Reference [96], statistically estimating the human role is not sufficient in various human-involved systems. Although ML techniques can reproduce human skills by building artificial models and algorithms and handle decision-making processes, the collaboration of humans and machines raise the mutual ML [97]. This type of ML focuses on the human self-learning and machine self-supervised learning capabilities and tries to capture, not only the information on machine status, but also human observations, in order to extract decision-making policies in human–machine integrated environments. Nevertheless, mutual ML leads to more complex and time-consuming operations for effectively managing the dynamic industrial processes in real-time. In this perspective, the challenges and potential of mutual human–machine learning in upcoming industrial areas were described in Reference [97] and illustrative collaboration scenarios were presented.

### 6.2. Implemented ML-Based Solutions

In Reference [98], a human-in-the-loop (HIL) approach was proposed and evaluated to determine the human operator’s choice complexity (OCC) in a manufacturing system, where a real human was encapsulated in smart manufacturing settings and OCC represented the selection of the proper component, for example, a tool, on a mixed model assembly line (MMAL) among a variety of components. In this approach, the required time interval for the component selection was related with the dynamics operator’s performance and was controlled by the number of options and the task complexity. The particular characteristics that defined and affected the OCC were initially identified and then, they were used to construct a regression model, where the human reaction time, as a function of the degree of choice complexity stood for the response variable (feedback) that trained several ML algorithms, that is, linear regression, regression trees, regression rules, instance-based learning algorithms, and SVMs. Among them, the supervised learning based on linear regression handled regression tasks by modeling a target prediction value based on independent variables and was fitted using the least squares. Also, the RandF method was exploited that use binary decision trees as a non-parametric supervised learning method for classification and regression and predicted target numerical values of variables represented in the leaf nodes. Besides, the regression rules were used and applied a step-wise selection, in order to obtain adequate attribute combinations with respect to a decision table. Additionally, instance-based learning algorithms were adopted, that is, the KNN regression method, and made a comparison between recent problem instances with those observed during training and stored in memory. Finally, SVMs with Gaussian kernels were developed via sequential minimal optimization algorithms. The aforementioned algorithms aimed at accurately predicting the cycle time according to the complexity of the tasks and the influence of CCO on the operator’s resultfulness. Overall, the performance of the linear regression method in terms of the correlation coefficient, the mean absolute error (MAE) and the root mean squared error (RMSE) was not satisfactory, whereas the RandF surpassed all the other algorithms.

In industrial applications, a long time is typically required, during the manufacturing phase, where a production system comes close to its desired operational performance. This phase is called ramp-up and is inherently dynamic and unpredictable, dramatically affecting the economic factors of an industrial system. Ramp-up strongly depends on the knowledge and expertise of the human operators. In Reference [99], the advantages of human–machine symbiosis during ramp-up were demonstrated by combining human intelligence in taking the best decisions and structured ramp-up experience with the machine’s capability for processing data quickly, accurately, and reliably, extracting optimal policies. Specifically, a Q-learning, that is, RL strategy guided by expert production engineers was proposed that minimized the unnecessary iterations in human decision-making processes, as well as the required data sets by conveniently acting for different ramp-up states. This learning approach exploited a specific update rule, that is, the update policy of a state value depending on new data entry, which involved the estimation of a new state value through a comparison procedure between current and previous state values. The validation of this strategy was obtained, using a ramp-up emulator and three existing and well-established machine-based exploration strategies as a reference, i.e, random exploration, greedy exploration, and greedy exploration with increased probability. In random exploration, the ramp-up actions are randomly selected to generate the data set. On the contrary, greedy exploration opts for actions that correspond to the highest possible reward by using a fixed probability, whereas greedy exploration with increased probability exploits a variable probability that depends on the number of iterations. The results indicated that, instead of exclusively using logarithmic machine-based random exploration, exploiting also the human knowledge was a more efficient strategy that closely approached the optimal behavior, while requiring significantly less data. On the other hand, the greedy exploration strategy enabled the generation of satisfactory results, but did not take advantage of the human element and hence, unnecessarily led to more states and implied a longer ramp-up time.

The integration of humans in a CPS through the assistance of human activity recognition (HAR), based on wearable sensors was studied in Reference [100]. In particular, a DL algorithm was used, accelerating the HAR process and facilitating the classification and analysis of daily human activities. More specifically, the DL-based CNN algorithm was adopted. The preprocessing of the data from wearable devices in different body positions in HAR tasks was a substantive component of this DL algorithm and included data segmentation and data transformation. Thus, the impact of different data segmentation methods on the DL performance was analysed and four data transformation approaches were compared, i.e, raw acceleration data, the multi-channel method, the spectrogram method, and the spectrogram integrated with shallow features method. The experimental results validated the classification accuracy performance of the CNN-aided HAR in different scenarios, when multiple wearable devices and single or multiple sensors were used. It was shown that the accuracy reached a value of 97.20% for eight daily activities, according to the data from seven wearable sensors, and it was demonstrated that CNN surpass by far other common ML methods, that is, ANNs, Decision Tree, KNN, Naive Bayes, and SVM. However, the accuracy of RandF was only 7.22% lower than CNN. These results also revealed that the multi-channel method performed better in both classification accuracy and training time, while the length of data segment drastically influenced the classification accuracy of the CNN-aided model. By increasing the segment length, the accuracy drastically improves.

Table 6 presents the main objectives of tackling HMI issues, as well as the employed ML solutions.

## 7. Open Issues

The latest advancements in the ML-inspired methods and the rise of DL have opened up new opportunities towards revolutionizing Industry 4.0 and beyond and have led to the possibility of realizing highly autonomous and effective industrial operations, while enhancing the fault diagnosis, mitigating human faults, safeguarding the security, and preventing unexpected losses, under complex and dynamic scenarios. Nevertheless, current work lays practical and methodological grounds for future research, since open research issues there exist and require attention. The range of challenges and future research directions can be summarized as follows.

### 7.1. Practical Challenges

The ML techniques depend, in principle, on massive and high-quality labeled data sets collected by sensors and network equipment, including manufacturing data collection and control systems that monitor the status of machines and radio frequency identification (RFID) that automatically identify and track tags attached to objects within factory halls, to obtain the prospective performance gain in the fault diagnosis field and attain robustness and reliability. As the IIoT emerges and cloud computing becomes widely adopted, data availability increases. However, processing large amounts of data in depth to find a near optimal solution, requires a huge computational effort. Nowadays, powerful multi-core central processing unit (CPU) architectures, graphics processing units (GPUs) and broad availability of libraries for DL (e.g., Reference [101]) allow for fast, parallel data processing for large-scale and real-time fault prognosis. With a view to real-time operation, field-programmable gate array (FPGAs) have shown stronger potential over GPUs in scenarios, where power consumption restrictions, flexibility to process with different data types, for example, binary, ternary and even custom ones, and performance in DNN comes into play massively [102]. Hence, upgrading CPS embedded electronics is necessary to enable the learning algorithm implementation on FPGAs.

Owing to the rapid development of hardware technology and computational power, ML and DL methods can be widely employed in industrial settings. Nevertheless, data-based modeling and fusion issues are usually observed, since the data is vulnerable to losses, redundancy, mislabeling, class imbalance, non-stationarity, and heterogeneity of information, which in turns hinders the effectiveness of the training procedure. Also, modification and enhancement of the ML framework to handle specific scenarios, including efficient error propagation and heuristic training regime, are not trivial tasks. To address the aforementioned challenging issues, future research should be devoted to the improvement of the computing efficiency and processing time of the learning algorithm and the design of learning methods, which effectively adapt to various degrees of uncertainty. Moreover, known fault types should be predefined and the ML methods should be modified to detect and recognize unknown fault conditions. In this direction, it is critical to realize more sophisticated data cleaning, in order to enhance the quality of the source data and subsequently meliorate the fault detection models. Targeted exploration, predefined experimentation, and incorporation of prior expert knowledge during the initialization of the learning models could reduce the number of required iterations and increase the quality of the learning policy. Future research could also include the application of parameter-optimisation techniques, such as genetic algorithms and particle-swarm optimisation. To further reduce the number of the required tasks, adding a confidence score to predictions and a scale factor to the generated actions is suggested. Furthermore, different ML techniques could be combined, in order to cooperatively accomplish the fault prediction procedure. Hence, the employment of hybrid and ensemble ML schemes is foreseen, considering the accuracy requirements, the acquisition of the communication parameters from the network elements, the centralized or distributed diagnostic performance, and the architecture design.

Existing results may not be able to be generalised in nature, since location-based restrictions may emerge and the empirical findings may be application-specific. Therefore, the ML-based methods should be applied to any other measured signals in industrial fields to verify whether the findings remain valid. Currently, there is a gap in acquiring data from diverse application scenarios. Thus, implementing test-beds and conducting real experiments in different industrial areas is indispensable, in order to validate the accuracy of the learning algorithms in terms of fault prediction, especially in scenarios with dynamically changing environments and increased latency and resiliency requirements. In order to maintain validity over time, it is recommended to periodically refresh and re-run the ML algorithms, using recent data.

Since ML-based Industry 4.0 stands for a comparatively young technology and research field, there is no unified perspective on what the ML technological advances imply. To eliminate barriers among various disciplines, new educational and specialized training strategies should be developed within industries, while increasing the level of cooperation between universities and industrial sectors and bringing together researchers and practitioners from different fields. In this respect, governments should also provide tax incentives to manufacturing companies, in order to promote the adoption of Industry 4.0 principles.

### 7.2. Networking, HMI, and Security Issues

Although the proposed frameworks and implementations can be potentially used in several industrial fields and processes, cloud and fog computing has significant limitations and further research work is required. For instance, the proposed approaches directly depend on the availability or deployment of physical devices in the factory, whereas there exist limited relevant libraries and platforms that support fog computing. Besides, fog nodes are characterised by modest computing capabilities, which prevents their use in certain ML applications with huge computation loads. Thus, multiple fog devices are required to enhance the running efficiency and obtain accurate and fast analytics. Additionally, it will be worthwhile to perform the classification regression experiments on other types of productions. Also, future work may focus on cloud-based parallel ML algorithms for big data management and analysis.

While current work has demonstrated the potential of incorporating humans in Industry 4.0 contexts using representative industrial scenarios, it partially captures the actual complexity in the decision-making processes. Therefore, it is indispensable to further validate the generic feasibility, applicability, and scalability of the proposed ML-based methods in full-scale industrial applications and human-involved systems, actual production systems, and real assembly lines with the proper resources. It is also envisaged to conduct inferential analysis to further assess the major differences amongst the presented approaches.

To combat ever evolving attack types against the safety of critical industrial infrastructures and the reliability of data transmission, including hijacking attacks targeting the data delivery node, damaging or stealing its load, advanced ML methods are needed. Current work can be extended by applying the intelligent mechanisms on more manufacturing processes of real Industry 4.0 systems and validating their performance in real-world cyber-physical environments and peculiar cases, such as complex malicious attack protection, data recovery, online learning, given the critical nature of those cases. Besides, extracting more than one type of training data is also required, in order to further enhance the accuracy of threat detection. Finally, as more IIoT devices are installed and connected, under a variety of abnormal conditions during the manufacturing process, both software and hardware security of IIoT devices and platforms should be considered in future works [103].

**Figure 4:**
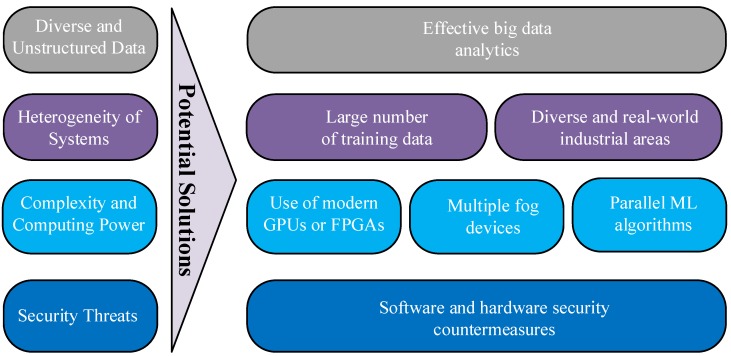
Open issues and potential solutions towards tackling faults in Industry 4.0 through ML.

As it has been observed from the previous discussion, the exist various remaining open issues for the efficient implementation and operation of ML in Industry 4.0 environments. Figure 4 depicts the current major open issues towards tackling faults in Industry 4.0 environments and potential solutions that ML-based methods should adopt.

## 8. Conclusions

The integration of artificial intelligence and machine learning in different sectors of the human society has played a key role in enabling exciting new possibilities in improving the quality of life and safeguarding the sustainability of our ecosystem. In the context of Industry 4.0, AI/ML enables a plethora of improvements throughout the manufacturing loop, optimizing processes, in terms of waste reduction, automation and product quality. A highly important area in realizing the Industry 4.0 vision is the timely detection and prevention of faults, leveraging the Industrial Internet of Things deployments and the subsequent acquisition and processing of heterogeneous big data. Towards this end, this survey presented relevant ML solutions, classifying them according to their learning process. Moreover, closely related aspects, necessary to provide a holistic overview of fault detection and prediction, that is, cyber-security issues and the role of humans within the manufacturing loop, were thoroughly discussed. Finally, aiming to foster further developments in this field, open issues were given, outlining the shortcomings of the current solutions and drawing possible future research directions. 

## Figures and Tables

**Table 1:** Relevant surveys and tutorials on artificial intelligence (AI)/ML and Industry 4.0.

Reference	Short Description	Scope of ML in Industry 4.0
Xu H. et al., 2018 [10]	CPS aspects in IIoT	IIoT, cloud/edge computing, sensing and decision-making
Rehman et al., 2018 [16]	The role of BDA in IIoT	ML-based BDA for self-organization and prediction
Wuest et al., 2016 [15]	ML’s role in manufacturing	Focus on supervised learning for fault diagnosis
Ge et al., 2017 [19]	Data mining and BDA in the process industry	Supervised, unsupervised and semi-supervised learning for BDA
Kim et al., 2018 [20]	Overview of ML in machining processes	Brief summary on tool-wear and condition monitoring
Xu L. D. et al., 2018 [21]	Interaction of big data and CPS in Industry 4.0	Nothing in particular
Wang et al., 2018 [23]	DL for smart manufacturing	Brief overview of fault detection and prediction
Ramotsoela et al., 2018 [25]	Overview of anomaly detection in IIoT	Presentation of implementation aspects
Aggour et al., 2019 [26]	AI/ML use cases for manufacturing and inspection	Presentation of specific applications for component inspection and life prediction
Weichert et al., 2019 [27]	ML for process optimization	Focus on production quality, brief overview of fault detection and prediction
Xu X. et al., 2017 [28]	Strategies for BDA, ontology modeling and deduction	Brief overview of DL for predictive diagnostics
Cheng et al., 2017 [29]	Data mining and BDA for production management	Nothing in particular
This survey	ML-based fault detection, prediction and prevention	Classification and analysis of ML for fault detection, predictive maintenance, HMI and security

**Table 2:** Target of cloud/fog/edge architectures and respective ML solutions for Industry 4.0.

Reference	Target of Relevant Architecture	ML Solution
O’Donovan et al., 2018 [32]	Minimisation of failures and latency reduction	SVM
Wan et al., 2018 [33]	Improvement of D2C and D2D communication and resource management optimization	General approach for various AI/ML solutions
Lee et al., 2017 [34]	Improvement of process quality and dynamic mixed data flow control	SVR, RBF, and DBL-DL
Li et al., 2018 [36]	Detection of defect type and degree	CNN
Lavassani et al., 2018 [37]	Reduction of spectrum usage and energy consumption	Distributed sensor learning
Sodhro et al., 2019 [38]	Sensing and processing execution time management and transmit power reduction	General approach for various AI/ML solutions
Wu et al., 2017 [39]	Latency reduction, reliability improvement and scalability provisioning	RandF

**Table 3:** Fault detection setting and respective ML solutions for Industry 4.0.

Reference	Fault Detection Setting	ML Solution
Maier et al., 2013 [41]	Automated detection for circuit boards	ANNs, SVMs and WMV
Jin et al., 2016 [43]	Missing syndromes due to fragmented repair logs	SVM, ANN, Naive Bayes, and Decision Tree
Mathew et al., 2018 [45]	Imbalanced data sets	WK-SMOTE SVM
Lin et al., 2019 [46]	Imbalanced data sets and concept drifts	Ensemble learning with various offline classifiers
Lee et al., 2016 [47]	Imbalanced data sets of semiconductor production	Comparison of three sampling- based, four ensemble, four instance- based, and two SVM methods
Syafrudin et al., 2018 [48]	Wide range of data types	DBSCAN-based RandF
Lei et al., 2016 [49]	Use of unlabeled data, lack of previous knowledge and diagnostic experience	Two-stage NN with sparse filtering and softmax regression
Yang et al., 2016 [50]	Use of raw vibration signals from wind turbines	Multiple hierarchical ELMs
Diaz-Rozo et al., 2017 [52]	Machine spindle monitoring	K-means, hierarchical, agglo- merative and Gaussian mixture
Pan et al., 2018 [54]	Use of noisy mechanical data	DL-based LiftingNet
Sohaib et al., 2017 [55]	Use of vibration acceleration signals for bearing and crack size identification	SAE-based DNNs.
Luo et al., 2019 [56]	Time-varying signal features and early fault detection	DL-based dynamic properties extraction
Tao et al., 2019 [57]	Failures in the gearbox of mechanical equipment	MGRU-based NN
Wen et al., 2019 [58]	Automatic range adjustment of the CLR scheduler	SECNN with MMCCLR
Iqbal et al., 2019 [59]	Use of multi-type spatial- temporal signals of an automotive assembly line	DL-based FDI with DAEs

**Table 4:** Predictive maintenance setting and respective ML solutions for Industry 4.0.

Reference	Predictive Maintenance Setting	ML Solution
Susto et al., 2015 [62]	Imbalanced data sets of integral type faults	MC supervised method
Yan et al., 2017 [64]	Unstructured multi- source heterogeneous data	Multi-scale analysis (envelope, time-frequency)
Wu et al., 2017 [65]	Heterogeneous data	RandF
Shin et al., 2018 [66]	Self-healing in shop-floor	SVM
Kuo et al., 2017 [67]	Low-complexity operation for SMEs	NN-based for online feature dimensionality reduction and automated prediction
Lin et al., 2017 [68]	Bias mitigation when using a single classifier type	Ensemble learning with MC types and diversity
Yu et al., 2019 [71]	Large-scale monitoring with unlabeled data	K-means, DPCA-based T-squared and SPE
Peres et al., 2018 [72]	Dynamic changes at the shop-floor level	K-means
Yan et al., 2018 [73]	Automated RUL prediction without experts’ knowledge	DL-based DECG
Sun et al., 2019 [74]	RUL relevant feature transfer in DL network	DTL with SAE
Cheng et al., 2019 [75]	Heterogeneous feature extraction from massive signals	AKSC with LSTM-RNN
Shi et al., 2019 [76]	Tool wear identification in ultra-precision manufacturing without experts’ knowledge	Feature spaces-based DL

**Table 5:** Target of security mechanisms and respective ML solutions for Industry 4.0.

Reference	Target of Security Mechanisms	ML Solution
Moustafa et al., 2018 [81]	Monitoring and detection of cyber-attacks in Industry 4.0	MHMM
Wu et al., 2019 [87]	Detection of cyber-physical attacks in 3-D printing processes	KNN, RandF, and anomaly detection
Park et al., 2018 [88]	Detection of anomalies in multi-variety production systems	DNN
Keliris et al., 2016 [89]	Detection of abnormalities and malicious activities	SVM

**Table 6:** Target of Human–Machine Interaction (HMI) implementations and respective ML solutions for Industry 4.0.

Reference	Target of HMI Implementation	ML Solution
Busogi et al., 2017 [98]	Prediction of cycle time with respect to task complexity	Linear regression, regression trees, instance-based and SVM
Doltsinis et al., 2018 [99]	Reduction of the required iterations and data sets	Q-learning
Zheng et al., 2018 [100]	Classification and analysis of daily human activities	CNN

## Abbreviations

The following abbreviations are used in this manuscript:

3-D	Three Dimensional
5G	Fifth Generation
AE	Auto-Encoder
AGV	Automated Guided Vehicle
AI	Artificial Intelligence
AKSC	Adaptive Kernel Spectral Clustering
ANN	Artificial Neural Network
AR	Augmented Reality
BDA	Big Data Analytics
BMM	Beta Mixture Model
BPNN	Back Propagation Neural Network
CC	Cloud Computing
CIM	Computer-Integrated Manufacturing
CLR	Cyclic Learning Rate
CMS	Cyber-Manufacturing System
CNC	Computer Numerical Control
CNN	Convolutional Neural Network
CPS	Cyber-Physical System
CWRU	Case Western Reserve University
D2C	Device-to-Cloud
D2D	Device-to-Device
DAE	Deep Auto-Encoder
DBSCAN	Density-Based Spatial Clustering of Applications with Noise
DDA	Deep Denoising Auto-Encoder
DDD	Diversity for Dealing with Drift
DECG	Device Electrocariogram
DL	Deep Learning
DNN	Deep Neural Network
DPCA	Distributed Principal Component Analysis
DTL	Deep Transfer Learning
EC2	Elastic Compute Cloud
ELM	Extreme Learning Machine
FDI	Fault Detection and Isolation
FPGA	Field-Programmable Gate Array
GPU	Graphics Processing Unit
GRU	Gated Recurrent Unit
HAR	Human Activity Recognition
HCPS	Human Cyber-Physical System
HIL	Human-in-the-Loop
HITL	Hardware-in-the-Loop
HMM	Hidden Markov Mechanism
HPS	Human Physical System
IaaS	intelligence-as-a-Service
ICA	Independent Component Analysis
ICS	Industrial Control System
IDARTS	Intelligent Data Analysis and Real-Time Supervision
IFS	Intelligent Factory Space
IIoT	Industrial Internet of Things
IIRA	Industrial Internet Reference Architecture
IoT	Internet of Things
IWSN	Industrial Wireless Sensor Network
KNN	K-Nearest Neighbor
LDA	Linear-Discriminate Analysis
LSTM	Long Short-Term Memory
M2M	Machine-to-Machine
MC	Multiple Classifiers
MGRU	Multilayer Gated Recurrent Unit
MHMM	Mixture-Hidden Markov Model
ML	Machine Learning
MLSTM	Multilayer Long Short-Term Memory
MMAL	Mixed Model Assembly Line
MMCCLR	Max-Min Cosine Cyclic Learning Rate Scheduler
MS	Manufacturing System
NN	Neural Network
OCC	Operator’s Choice Complexity
PCA	Principal Component Analysis
Pi	Patented Intelligence
PLC	Programmable Logic Controller
PMLL	Predictive Modelling Markup Language
RAMI 4.0	Reference Architecture Model Industry 4.0
RandF	Random Forest
RBF	Radial Basis Function
RBM	Restricted Boltzmann Machine
RFID	Radio Frequency Identification
RL	Reinforcement Learning
RNN	Recurrent Neural Network
RUL	Remaining Useful Lifetime
RVM	Relevance Vector Machine
SAE	Stacked Auto-Encoder
SECNN	Snapshot Ensemble Convolutional Neural Network
SGD	Stochastic Gradient Descent
SME	Small and Medium-sized Enterprise
SMOTE	Synthetic Minority Oversampling Technique
SPE	Square Predicted Error
SVM	Support Vector Machine
SVR	Support Vector Regression
VR	Virtual Reality
WK	Weighted Kernel
WMV	Weighted-Majority Voting
